# Combined microbiological approach to screening of producers of proteases with hemostasis system proteins activity among micromycetes

**DOI:** 10.1016/j.btre.2018.e00265

**Published:** 2018-06-18

**Authors:** Alexander A. Osmolovskiy, Anna A. Lukianova, Elena S. Zvonareva, Valeriana G. Kreyer, Nina A. Baranova, Nikolay S. Egorov

**Affiliations:** a Department of Microbiology, Faculty of Biology, Lomonosov Moscow State University, Moscow, Russian Federation; b Faculty of Biotechnology, Lomonosov Moscow State University, Moscow, Russian Federation; c International Biotechnological Center, Lomonosov Moscow State University, Moscow, Russian Federation

## Abstract

A scheme for screening of micromycetes - producers of proteases with the activity of hemostasis system proteins, based on their enzymatic indices determination and the activity towards chromogenic peptide substrates for proteins of the hemostasis system was developed. Depending on the ability of proteases producers to cleave such substrates, an enzymatic reaction in conditions containing human plasma is suggested, which makes it possible to identify the potentiality of the target plasma hemostasis proenzymes activation.

## Highlights

• Most of micromycetes are produced proteolytic enzymes which can affect human hemostasis proteins.
• For determination of such activity special chromogenic peptide substrates are used.
• Proteases secretion by micromycetes depends on medium composition.

## 1 Introduction

One of the main challenges of modern medicine is the diagnosis and treatment of thrombotic complications. Various thrombolytic agents, which are used in therapy, are proteolytic enzymes with the activity similar to the one of hemostasis system proteases [1]. Such activity was also found in proteases present in venom of snakes and in cultures of microorganisms. The preparations obtained on their basis are used as a part of diagnostic kits and as therapeutic agents for the detection and treatment of such complications [[2], [3], [4], [5]].

Extracellular proteolytic enzymes of micromycetes from different ecological groups have the ability to act on hemostasis system proteins as activators (triggering procoagulant and anticoagulant processes) or direct fibrinolytic agents (breaking down already formed thrombi) [4,6]. But there are no unified ways to determine their ability to produce such proteases.

## 2 Materials and methods

Micromycetes for screening were supplied by the Department of Microbiology, Moscow State University. The strains were maintained in tubes with slant agar. Agar-plate cultivation of the fungi was carried out on Czapek’s media with 1% casein or fibrin addition as the single source of nitrogen. After 5 days of cultivation at 28 °C, 5 ml of 0.08% Coomassie brilliant blue G-250 in 3.5% perchloric acid were poured into Petri dishes and the ratio of the hydrolysis zone diameter to the colony zone diameter was measured [7].

Submerged cultivation (in shaking flasks 750 mL in volume with 100 mL of the nutrient media using orbital shakers, 200 rpm, 28 °C) was carried out in two stages, first, on the medium containing wort, glucose, and pepton [8], and then, after two days of cultivation, the biomass was transferred into the fermentation media containing (g/L) glucose, 30.0; glycerol, 70.0; NaNO_3_, 2.0; fish flour 5.0; MgSO_4_ ⋅ 7H_2_O, 0.5; and KH_2_PO_4_, 0.5 (medium 1) or glucose, 35.0; starch, 1.2; peptone, 5.0; fish flour 5.0; NaCl, 2.0; MgSO_4_ ⋅ 7H_2_O, 0.5; and KH_2_PO_4_, 0.5 (medium 2).

The activity of extracellular proteases towards hemostatic proteins was assessed in the culture medium after preliminary filtration of the biomass. Amidolytic activity towards chromogenic peptide substrates was determined by adding 0.05 M Tris-HCl buffer (pH 8.2) and substrate to the culture fluid as shown earlier [9]. The activator activity was evaluated by the cleavage of the above mentioned chromogenic peptide substrates after preliminary enzyme incubation with plasma (instead of a buffer in the reaction mixture). Optical absorption of the resulting solutions was measured at 405 nm. The quantity of *p*-nitroaniline (μmol) cleaved from the substrate in 1 mL of the sample solution per 1 min was taken as 1 unit (U) of activity.

The testing was carried out using chromogenic peptide substrates cleaved by various proteins of the human hemostasis system: H-D-Val-Leu-Lys-pNA, a plasmin substrate; Bz-Ile-Glu(OR)-Gly-Arg-pNA and Z-D-Arg-Gly-Arg-pNA, human plasma Xa factor substrates; Glp-Pro-Arg-pNA, a substrate for activated protein C; Glp-Gly-Arg-pNA, an urokinase substrate; H-D-Phe-Pip-Arg-pNA and Tos-Gly-Pro-Arg-pNA, thrombin substrates.

Experiments were carried out in three replicates, and the values presented are averages with the error not exceeding 5–7%.

## 3 Results

Twenty micromycete cultures of *Aspergillus, Cladosporium, Paecilomyces, Purpureocillum,* and *Tolypocladium* genera were tested for the capacity to produce proteases affecting human hemostatic proteins.

The activity of hemostatic proteases can be determined by the cleavage of sensitive chromogenic substrates, *p*-nitroanilides, after preliminary incubation in the presence of protein activators.

**Fig. 1:**
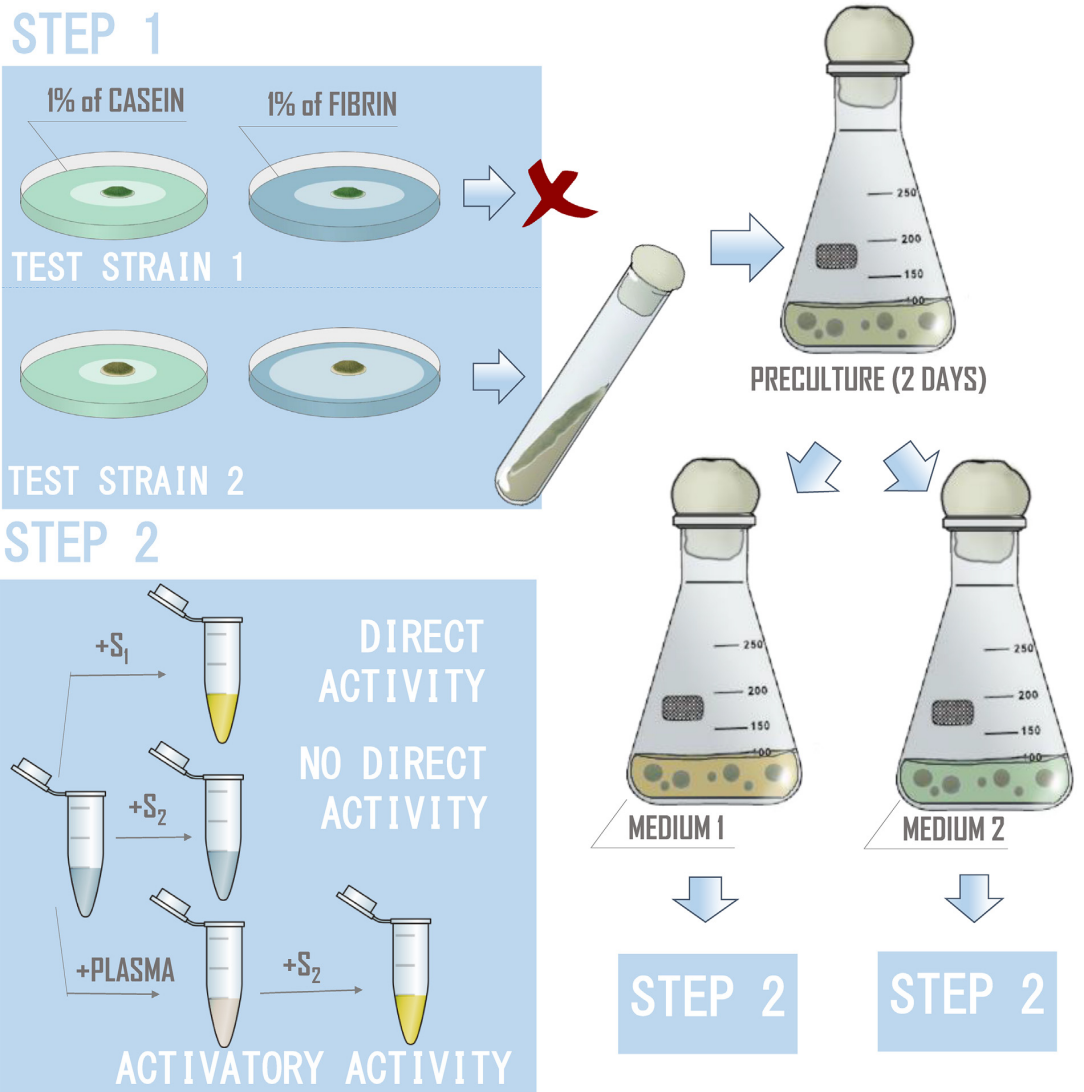
An experimental scheme for the evaluation of hemostatically active proteases, presumably produced by micromycetes.

An experimental scheme for testing the activity of proteases obtained from the fungal culture medium is based on this principal. Initially, micromycetes were selected according to the productivity of protease production, calculated from the enzymatic indices (EI, the ratio between the hydrolysis zone diameter and the diameter of the colony grown on proteins containing media in Petri dishes), as indicated in Fig. 1. After this primary screening, cultures with higher EI for fibrin containing medium (values of EI more than 1.00 ± 0.05) and lower values for casein containing medium (values of EI less than 1.50 ± 0.1) were selected for further research.

Afterwords, a secondary screening with the determination of hemostatic proteins-like activity was carried out with the cultivation of micromycetes under submerged conditions, as indicated in Fig. 1. The direct activity towards a chromogenic peptide substrate, for instance S1 (as indicated in Fig. 1), was studied without the addition of human blood plasma into the incubation mixture. The activator activity was measured with the addition of human blood plasma into the incubation mixture if the activity of the culture medium towards another chromogenic substrate, for instance S2, another chromogenic substrate, was not detected. The fact of the presence of the activator activity towards the S2 substrate may imply the reaction of limited proteolysis of plasma hemostasis proteins by micromycete’s proteinases. The activator activity of fungal proteases towards the vital hemostatic proteins – protein C, factor X, plasminogen and prothrombin, was detected on the basis of the following hypothesis: if extracellular micromycete proteases can activate the corresponding plasma proteins, the activation products – activated protein C, factor Xa, plasmin and thrombin, will cleave their specific chromogenic substrates, *p*-nitroanilides, at the arginine residue to release free *p*-nitroaniline into the solution. The significant manifestation of one or another micromycetes extracellular proteases activity lay in the value range 33.5–71.5 U×10^−3^, the trace activity had values less than 9.5 U×10^−3^. It should be noted that not all the cultures studied produced proteases that were active in all of the reactions performed. Consequently, this approach makes it possible to identify the producers of proteases with the required activity among micromycetes.

It was thus possible to select 5 highly active protease-producing strains with the activity of hemostasis system proteins: *Aspergillus ochraceus* L-1 (a producer of proteases - activators of protein C and factor X), *Aspergillus terreus* 2 (a producer of proteases - prekallikrein activators), *Aspergillus ustus* 1 and *Cladosporium cladosporioides* 1 (producers of fibrinolytic enzymes) and *Purpureocillium lilacinum* k1 (a producer of thrombin-like proteases).

## 4 Discussions

The proposed approach involves two successive steps: cultivation of micromycetes in Petri dishes with globular (casein) and fibrillar (fibrin) protein substrates to reveal general proteolytic activity, and submerged cultivation on two media to determine the target activity.

Surface cultivation on media with hydrolysable substrates allows to determine the specificity of secreted proteases towards fibrillar proteins and to select the most active producer according to the value of the EI. Thus, producers were selected, the proteinases of which exhibit the least nonspecific proteolytic activity.

Submerged cultivation on media with and without a mineral nitrogen source allows to take into account a mixed type of nitrogen metabolism in mycelial fungi and fully gives an idea of the ability to produce extracellular proteases with the desired activity. The determination of a specific extracellular proteolytic activity in the culture fluid towards chromogenic peptide substrates for the hemostasis system proteins makes it possible to identify both the presence of the activity itself and to select the most active producer of such enzymes. Due to the proposed method, it was possible for the first time to detect the ability of micromycetes to produce proteases that activate some proteins of plasma hemostasis.

The screening strategy was developed on the basis of previous studies to determine the ability of proteases of micromycetes to affect the proteins of the hemostasis system [6,9]. The proposed strategy is aimed at identifying target activities already at the screening stage, and not with the isolation of enzymes, as is common [[10], [11], [12]]. Known ways for selection of protease producers include, as a rule, submerged cultivation of micromycetes, followed by analysis in the culture liquid of activity against proteins of the hemostasis system (fibrinolysis, clotting of plasma components etc.) and subsequent isolation of enzymes with determination of their substrate specificity. The developed strategy allows to select producers among micromycetes of proteases, highly active in relation to proteins of the human hemostasis system and having a certain specificity to them. In addition, the strategy combines ways to find out producers of proteases with both direct and activating effect on the proteins of the hemostasis system.

## Conflict of interest statement

There are no conflicts of interest with respect to any of the authors.
